# Prolyl Oligopeptidase Inhibition Attenuates Steatosis in the L02 Human Liver Cell Line

**DOI:** 10.1371/journal.pone.0165224

**Published:** 2016-10-19

**Authors:** Da Zhou, Bing-Hang Li, Jing Wang, Yong-Nian Ding, Yan Dong, Yuan-Wen Chen, Jian-Gao Fan

**Affiliations:** 1 Department of Gastroenterology, Xinhua Hospital Affiliated to Shanghai Jiao Tong University School of Medicine, Shanghai, China; 2 Department of Gastroenterology, the Second Affiliated Hospital of Xinjiang Medical University, Urumqi, China; 3 Department of Endocrinology, Xinhua Hospital Affiliated to Shanghai Jiaotong University School of Medicine, Shanghai, China; Laval University, CANADA

## Abstract

**Background:**

Prolyl oligopeptidase (POP) is a serine endopeptidase that is widely distributed *in vivo*, particularly in the liver. Significant changes in functional mitochondrial proteins involved with mitochondrial oxidoreductases/transporters and nucleic acid binding proteins were observed after POP inhibition in the liver, which suggested a role of POP in regulating liver energy metabolism. Steatosis in nonalcoholic fatty liver disease (NAFLD) is associated with disturbances in lipid and energy metabolism in hepatocytes. Here, we aimed to study the effect of POP on hepatocyte steatosis.

**Methods:**

The human liver cell line L02 was used to investigate the biological effects of POP. An *in vitro* cell model of steatosis was successfully induced with oleic acid and palmitic acid. L02 cells were also subjected to S17092 (a POP inhibitor) at different concentrations for 24 or 48 h. Ac-SDKP levels and POP activity were measured to assess the rate of inhibition of POP by S17092. The POP gene and protein expression levels were detected using real-time PCR and Western blots, respectively. Oil red O staining was performed and the triglyceride levels in the L02 cells were also measured. Cell proliferation and apoptosis were detected using CCK-8 and flow cytometry, respectively. The expression of genes involved in lipid metabolism was detected using real-time PCR. The effects of POP inhibition on LC3B II were detected by Western blot.

**Results:**

Compared with the control, the POP mRNA levels increased by approximately 30%, and the POP protein levels increased by almost 60% in the steatotic L02 cells. After S17092 (0.026~130 μM) incubation for 24 or 48 h, cell proliferation was significantly decreased in the free fatty acid (FFA)-treated cells at 26–130 μM; however, S17092 did not affect the proliferation of L02 cells after 24 h of incubation with S17092 at 0.026–65 μM without FFA treatment. S17092 treatment (13 and 26 μM) also elicited no significant effect on apoptosis in normal L02 cells, but FFA treatment increased cell apoptosis, which was attenuated by S17092 incubation. S17092 treatment inhibited intracellular POP activity and decreased the AcSDKP level at the concentration of 0.026–26 μM. After treatment with FFA for 24 h, oil red O staining revealed significant lipid accumulation in the cells in the model group compared with the controls; however, lipid accumulation was suppressed after the administration of S17092 (13 and 26 μM). Accordingly, the triglyceride levels in the FFA-treated cells were approximately 5-fold greater than those of the controls and were decreased by approximately 25% and 45% after the administration of S17092 at 13 and 26 μM, respectively. The mRNA levels of FASN, PPAR-γ, and SREBP-1c were higher in the FFA-treated cells than in the normal controls, and all of these levels were significantly inhibited in the presence of S17092 at both 13 and 26 μM. S17092 treatment did not affect LC3B II in the FFA-treated cells compared with FFA treatment alone.

**Conclusion:**

The expression of POP increases with hepatocyte steatosis, and POP inhibitors can significantly reduce intracellular lipid accumulation, which might be related to the inhibition of genes involved in lipid synthesis.

## Introduction

Nonalcoholic fatty liver disease (NAFLD) is a chronic metabolic disorder of the liver, which has become the most common liver disease in developed countries, including China [1]. NAFLD consists of a histological spectrum ranging from simple steatosis to nonalcoholic steatohepatitis (NASH), advanced fibrosis, and cirrhosis and eventually leads to liver failure or hepatocellular carcinoma [2]. The pathogenesis of NAFLD is complex and includes abnormal fat metabolism and hormone secretion, environmental and genetic factors, oxidative stress and lipid peroxidation damage, and immune-related reactions [3–5]. These mechanisms are not well understood, and effective measures for preventing and treating NAFLD are lacking.

Prolyl oligopeptidase (POP, EC 3.4.21.26), also known as prolyl endopeptidase (PEP), is thought to be a serine endopeptidase that hydrolyzes proline-containing peptides shorter than 30 amino acids (pro-Xaa, where X is any amino acid except pro) specifically at the carboxyl terminal of internal proline residues. A variety of hormones and cytokines have been suggested to be substrates of this enzyme [6, 7]. In the liver, the quantities of POP are different between mice and humans, but its activity is relatively high in both mice and humans [8, 9]. POP is present in the cytoplasm and nuclei of hepatocytes; however, the biological effects of POP in the liver have not been well elucidated [10–12].

Hepatic steatosis is characteristic of the onset of fatty liver disease, and it refers to an imbalance of the energy metabolism of liver cells, lipid metabolism, and proinflammatory and anti-inflammatory cytokine secretions. A recent study of the effects of POP inhibition in the liver revealed predominant changes in the mitochondrial oxidoreductase/transporter as well as in nucleic acid binding proteins, which suggests a role of POP in the regulation of liver energy metabolism [10] and thus in hepatocyte steatosis. Here, we aimed to explore the effects of POP on hepatocyte steatosis using the human hepatocyte cell line L02 *in vitro*.

## Materials and Methods

### Cell culture

Human L02 hepatocytes were obtained from the Chinese Academy of Science (Shanghai, China). The cells were cultured in 1640 culture medium (GIBCO, Grand Island, NY) supplemented with 10% fetal bovine serum (FBS, GIBCO, Grand Island, NY) and incubated at 37°C in a humidified atmosphere of 5% CO_2_. To establish a cell model of fat overloading, palmitic (C16:0) and oleic (C18:1) acids (Sigma, St. Louis, MO; free fatty acids, FFAs) were added into the culture solution at a final concentration of 0.5 mM at a 1:2 ratio, and the cells were cultured for 24 h [13].

### POP inhibitor treatment

The pharmacological inhibitor of POP activity S17092 used in this study (Sigma, St. Louis, MO) [14]. S17092 is a highly potent (Ki = 1.5nM), specific and cell permeant inhibitor of human POP[15]. The L02 cells were divided into control, model (FFA only) and treatment groups (FFA+S17092). The cells were treated with increasing concentrations of S17092 (0.026, 0.26, 2.6, 13, 26, 65, 130 μM, approximately 0.01, 0.1, 1, 5, 10, 25, or 50 μg/ml) or vehicle (dimethylsulfoxide, DMSO) for 24 h. The FFA mixtures and S17092 were added into the culture solution at the same time.

### Oil red O staining

Equal amounts of cells were plated on 3.5-cm dishes (approximately 1.5×10^5^ cells), and the cells were treated with POP inhibitor for 24 h. The cells were washed three times with D-PBS (FBS, GIBCO, Grand Island, NY) before being fixed with 4% paraformaldehyde for 15 minutes. After washing, 60% isopropanol was added, and the cells were allowed to stand for 5 minutes. Then, the cells were stained with freshly diluted oil red O solution for 15 minutes. The cells were washed twice with 60% isopropanol for 1 minute, rinsed with water and counterstained with hematoxylin for 30 seconds. Images were captured using a microscope (Leica DMI3000B, USA).

### Triglyceride (TG) assay

Cells from the different groups were harvested and washed twice with D-PBS. The intracellular triglycerides were measured using a triglyceride assay kit according to the manufacturer’s instruction (Applygen Technologies Inc., Beijing, China). The TG concentrations were normalized to the total cell protein concentration [16, 17].

### Cytotoxicity and cell proliferation assay

Cell death was evaluated colorimetrically by the activity of the lactate dehydrogenase (LDH) released in the media, using cytotoxicity LDH Assay Kit (Dojindo, Shanghai, China). Briefly, cells were treated with increasing concentrations of S17092 (0.026–130 μM) or vehicle (dimethylsulfoxide, DMSO) for 24 or 48 h, the liquid supernatant were collected and LDH activity was detected according to the manufacturer’s instruction; the absorbance (optical density, OD) at 490 nm was detected using a microplate reader (uQuant, Biotek, Winooski, VT). The average absorbance from each triplicate set of wells were calculated and subtract the background control value from each absorbance one and the percent cytotoxicity was expressed as a percentage of maximal reaction, which was obtained through the lysis of all cells. Cytotoxicity (%) = (sample OD − control OD) / (maximal reaction OD − control OD) ×100.

The proliferation rate of the L02 cells was determined using a cell counting kit-8 (CCK-8, Dojindo, Shanghai, China) according to the manufacturer’s instructions. In brief, L02 cells were seeded on a 96-well plate at the density of 10^4^ cells per well. Then, the cells were treated with increasing concentrations of S17092 (0.026–130 μM) or vehicle (dimethylsulfoxide, DMSO) for 24 or 48 h [15, 18]. CCK-8 was added, and the absorbance (optical density, OD) at 450 nm was detected using a microplate reader (uQuant, Biotek, Winooski, VT).

### Apoptosis analysis

Cell apoptosis was assessed using an Annexin V-FITC Apoptosis Detection Kit (Becton-Dickinson, San Jose, CA) according to the manufacturer’s instructions. Treated cells (1×10^6^ cells/ml) on 3.5-cm dishes were harvested and then stained with Annexin V and FITC at room temperature for 15 minutes. The apoptotic cells were detected with a FACScan flow cytometer, and the data were analyzed using CellQuest software (Becton-Dickinson, San Jose, CA).

### Real-time quantitative polymerase chain reaction (qPCR)

Total RNA was extracted from the treated cells using TRIzol (D9108B, Takara, Dalian, China) and reverse-transcribed into cDNA using PrimeScript RT Master mix (RR036A, Takara, Dalian, China). Real-time qPCR was performed using an Applied Biosystems 7500 real-time PCR system and SYBR Premix Ex Taq (Tli RnaseH Plus) (RR420A, Takara, Dalian, China). The primers for the target genes were synthesized by Sangon Biotech (Shanghai, China). The primer sequences used are listed in Table 1. Primer specificity was confirmed using dissociation curves created with the SDS software of the 7500 system. Actin was used as the internal control. Relative fold changes in gene expression were determined by the 2^−ΔΔCt^ method.

**Table 1:** Primers used for real-time PCR.

Gene	Forward primer	Reverse primer
Actin	5'-TCCTTCCTGGGCATGGAGT-3'	5'-CAGGAGGAGCAATGATCTTGAT-3'
FASN	5'-ATCCTACGCTCCGATGAGG-3'	5'-TCACAAACGAATGGACGATG-3'
PPAR-γ	5'-CCACATTACGAAGACATTCCA-3'	5'-CAGGCTCCACTTTGATTGC-3'
SREBP-1c	5'-ACGGGAGGATGGACTGACTT-3'	5'-AGGCTTCTTTGCTGTGAGATG-3'
POP	5’-CATCTCCCAAGAGGCTGACTA-3’	5’-GGGCAATAACACAACCAAAGA-3’

*Note*: FASN, fatty acid synthase; POP, prolyl oligopeptidase.

### Western Blot

Treated cells were lysed in ice-cold radio-immunoprecipitation assay (RIPA) buffer containing protease and phosphatase inhibitors (phenylmethylsulfonyl fluoride, PMSF) (Beyotime, Shanghai, China). The total protein was measured using a bicinchoninic acid protein assay (BCA, Beyotime, Shanghai, China). Rabbit anti-POP and rabbit anti-autophagosome marker microtubule-associated protein light chain 3B (LC3B) were purchased from Abcam (San Francisco, USA), and anti-actin was purchased from Beyotime (Shanghai, China). The western blot analyses were performed as previously described [19]. Immune complexes were detected using Immobilon Western Chemiluminescent HRP substrate (Millipore Corporation, Billerica, MA). The bands were quantified using Image Lab Version 2.0.1 (Bio-Rad, Hercules, CA). Actin was used as a loading control.

### POP activity and Ac-SDKP concentration assay

The POP activity assay was performed as described previously [15, 19]. Equal amounts of cells were plated on 3.5-cm dishes (approximately 5×10^5^ cells) and treated with increasing concentrations of S17092 (0.026–130 μM) for 2 h. Then cells were homogenized in 50 μl assay buffer (10 mmol/L Tris − HCl buffer, pH 7.4), centrifuged at 16,000g for 20 min at 4°C. The supernatant was incubated with 37.5 μl of assay buffer for 30 min at 37°C. After that, 2.5 μl of substrate (4 mmol/L Suc-Gly-Pro-AMC, Bachem) was added. The reaction was allowed to proceed for 60 min at 37°C and terminated by the addition of 50 μl of 1 mol/L sodium acetate buffer (pH 4.2). The formation of AMC was determined by measuring the fluorescence intensity at 460 nm with excitation at 355 nm using a Synergy^™^ H4 plate reader (BioTek Instruments, USA). Free AMC (Bachem AG, Bubendorf, Switzerland) was used as standard and the basal fluorenscence of Suc-Gly-Pro-AMC was subtracted.

Intracellular N-acetyl-seryl-aspartyl-lysyl-proline (Ac-SDKP) was measured using an enzyme immunoassay kit (SPI Bio and CEA, France) that was modified for the cells [19, 20]. Briefly, the cells were lysed with RIPA buffer containing 10 μmol/L captopril and 1 mmol/L PMSF. The cell total proteins of the different groups were determined by BCA, and the concentrations of Ac-SDKP were adjusted to the total proteins. The lysates were centrifuged at 14000 g for 10 min, and the supernatants were extracted with methanol. The samples and standards were then processed according to the manufacturer’s instructions.

### Statistical analysis

The results are expressed as the means ± the standard errors of the mean (SEMs). At least three independent experiments were performed for each experiment. The data were analyzed using Student’s t-tests or one-way analyses of variance (ANOVAs) followed by Mann Whitney U tests using the SAS software (release 8.02 TS Level 02 M0, NC, USA). *P* < 0.05 was considered statistically significant.

## Results

### POP mRNA and protein levels increased in the fat-overloaded L02 cells

**Fig 1:**
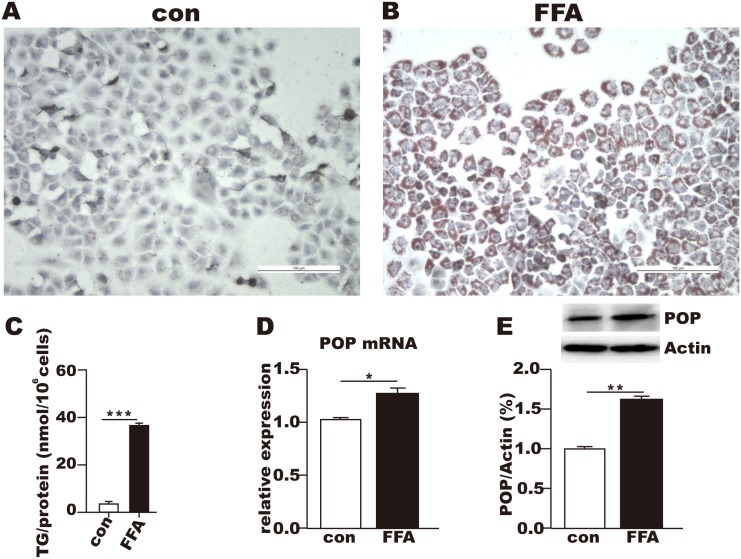
L02 cell steatosis and POP expression after FFA treatment for 24 h. (A-B) Oil red O staining of the control (A) and free fatty acid-treated cells (B). (C) Triglyceride concentrations in the control and FFA-treated cells. (D-E) POP mRNA expressions (D) and protein levels (E) in the control and FFA-treated cells. CON: control; FFA: free fatty acids; TG: triglyceride. * *P* < 0.05, ** *P* < 0.01, *** *P* < 0.001.

After treatment with the FFA mixtures (P:O) for 24 h, oil red O staining revealed that the lipid content significantly increased in the FFA-treated cells (Fig 1A and 1B); the TG level in the FFA-treated cells was almost 5 times greater than that of the control L02 cells (Fig 1C). POP mRNA was elevated by approximately 30% in the FFA-treated cells compared with the control cells (Fig 1D; *P* < 0.05), and the POP protein levels increased by almost 60% in the steatotic L02 cells compared to the level in the control cells (Fig 1E; *P* < 0.05).

### Effects of the POP inhibitor on cell proliferation and apoptosis

**Fig 2:**
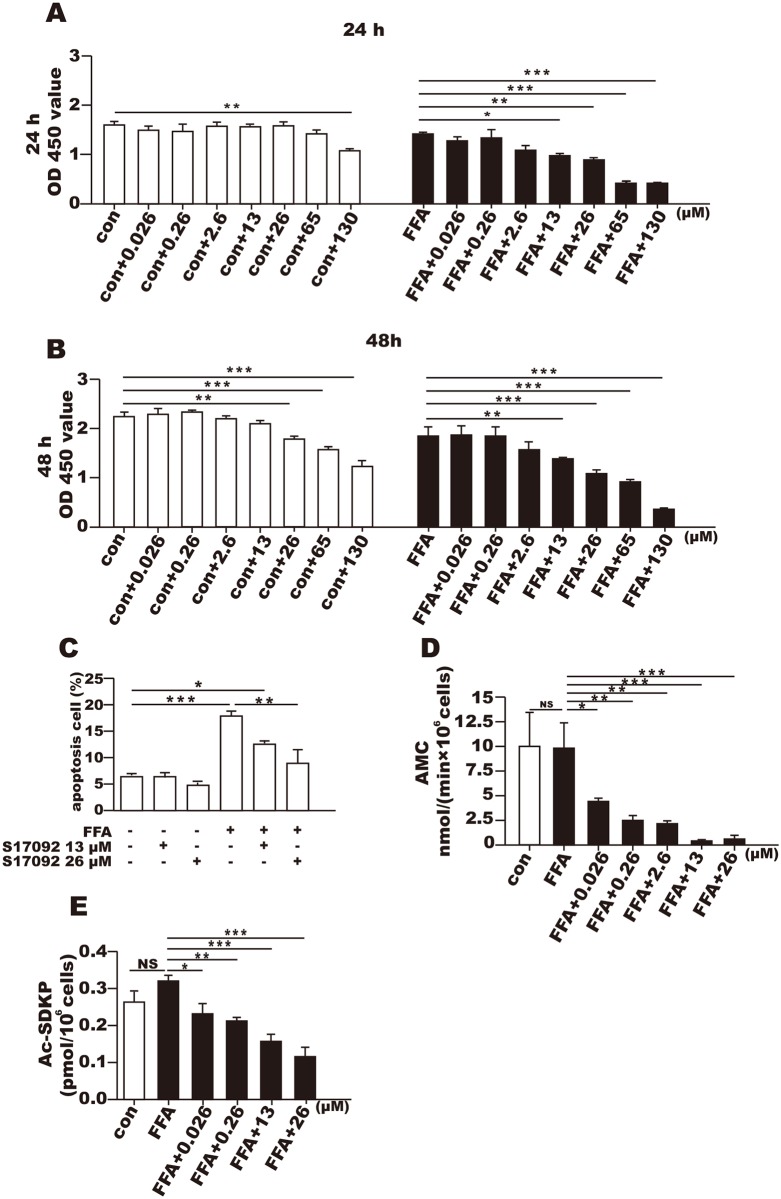
Effects of the POP inhibitor S17092 on L02 proliferation and apoptosis with and without FFA. (A) The proliferation of L02 cells treated with 0.026–130 μM S17092 with or without FFA for 24 h. (B) The proliferation of L02 cells treated with 0.026–130 μg/ml S17092 with or without FFA for 48 h. (C) The apoptosis rates of the L02 cells treated with S17092 with or without FFA for 24 h. (D) Intracellular POP activity in the control and FFA-treated L02 cells after incubation with S17092 at 0.026–26 μM for 2 h. (E) The intracellular AcSDKP level in the control and FFA-treated L02 cells after incubation with S17092 at 0.026–26 μM for 24 h. * *P* < 0.05, ** *P* < 0.01, *** *P* < 0.001.

The LDH activity in the supernatant did not significantly differ from corresponding control after S17092 (0.026~130 μM) incubation either for 24 or 48 h(S1A and S1B Fig). After incubation with S17092 (0.026~130 μM), cell proliferation was significantly decreased at only the concentration of 130μM after 24 h, but proliferation was also decreased at 26–130μM after 48 h of incubation. In the cells that were treated with FFA, cell proliferation was significantly inhibited by S17092 at the concentrations of 26–130μM at both 24 h and 48 h but not at lower concentrations (Fig 2A and 2B). S17092 did not affect the apoptosis of L02 cells in the absence of FAA when compared to apoptosis in the normal control cells (Fig 2C). However, FFA alone increased cell apoptosis, and S17092 attenuated FFA-induced cell apoptosis (Fig 2C; *P* < 0.05). S17092 treatment significantly inhibited intracellular POP activity at the concentrations of 0.026–26 μM after 2h incubation (Fig 2D); the intracellular level of AcSDKP was decreased after S17092 incubation for 24 h (Fig 2E; *P* < 0.05).

### The POP inhibitor attenuated intracellular lipid accumulation and suppressed the expression of lipid metabolism-associated genes without affecting the autophagy marker LC3B II in the L02 cells

**Fig 3:**
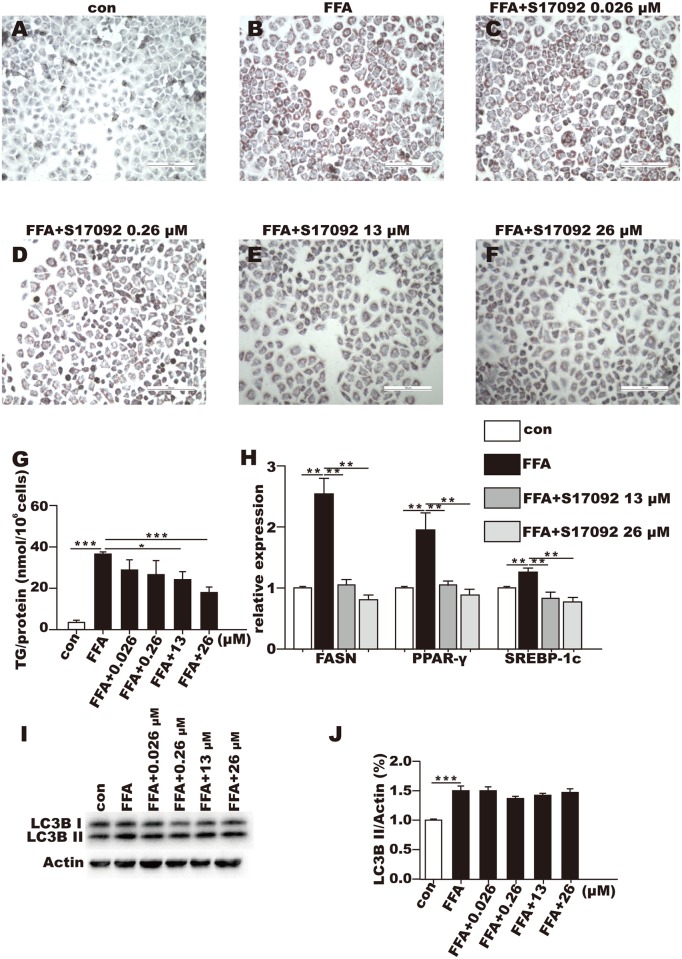
Expressions of genes involved in lipid metabolism in L02 cells with or without POP inhibition by S17902. (A-F) Oil red O staining of control cells (A), FFA-treated cells (B), cells treated with FFA+ S17902 (0.026 μM), (C) FFA+ S17902 (0.26 μM), (D) FFA+ S17902 (13 μM), (E) or FFA+ S17902 (26 μM) (F) for 24 h; (G) TG concentration in the L02 cells treated with S17092 for 24 h.(H) FASN/PPAR-γ/SREBP-1c mRNA expression levels in the controls and FFA-treated L02 cells with or without S17092 incubation for 24 h. (I-J) LC3B II protein expression levels in the controls and FFA-treated L02 cells with or without S17092 incubation for 24 h. * *P* < 0.05, ** *P* < 0.01, *** *P* < 0.001.

Treatment with S17092 (13 and 26 μM) for 24 h attenuated lipid accumulation in the FFA-treated cells as indicated by spotty oil red O staining, in contrast with the more intense pattern observed in the FFA-treated cells. Treatments with the lower concentrations of S17092 (0.026 and 0.26 μM) resulted in no significant inhibition (Fig 3A and 3F). Accordingly, TG was significantly decreased by approximately 25% and 45% in the FFA-treated cells after the administration of 13 and 26 μM S17092, respectively (Fig 3G; *P* < 0.05), but the changes did not reach statistical significance at the 0.026 or 0.26 μM concentrations. The expression levels of fatty acid synthase (FASN), peroxisome proliferator-activated receptor-γ (PPAR-γ), and sterol regulatory element-binding protein-1c (SREBP-1c) were significantly increased in the FFA-treated cells compared with the levels in the control cells. However, all of these genes were remarkably suppressed in the FFA-treated cells after treatment with S17092 at both 13 and 26 μg/ml (Fig 3H; *P* < 0.05). LC3B II in the FFA-treated L02 was were significantly increased after 24 h compared with the control cells, however, LC3B II in the FFA-treated cells was not affected by S17092 treatment (Fig 3I and 3J).

## Discussion

Here, we reported an increase in POP in steatotic L02 cells induced by FFA. Furthermore, the inhibition of POP by the specific inhibitor S17092 resulted in a significant inhibition of cell proliferation and an attenuation of lipid accumulation in the FFA-treated L02 cells that were accompanied by the downregulation of genes involved in lipid metabolism.

In contrast to the central nervous system, the physiological functions of POP in peripheral tissues have not been extensively investigated [6, 21, 22]. Studies have demonstrated that POP is present in both the cytoplasm and nuclei of liver cells [8]; POP activity is increased in the livers of newborn mice undergoing growth and maturation and during liver regeneration and repair after resection, but both of these physiological or pathological processes are blunted after POP inhibition by a synthesized specific POP inhibitor [11, 12, 23]. Here, our study found that both POP mRNA and protein were increased in steatotic hepatocytes. POP inhibition by S17092 (13–65 μM) successfully inhibited the proliferation of FFA-treated cells but not the control cells at 24 h. However, inhibitory effects were also observed in the control cells at 26–130 μM after 48 h of incubation. The mechanisms of POP in liver regeneration, repair and hepatocyte proliferation have not been clarified. A later study using a POP inhibitor reported predominant changes in the mitochondrial oxidordeuctase/transporter, which may affect ATP synthesis, as well as nucleic acid binding proteins, all of which suggested a role of POP in regulating liver energy metabolism [10]. The utilization of substrates and the regulation of energy metabolism are fundamentally important for cell proliferation, differentiation and maturation [24]. Therefore, it is reasonable to speculate that POP in the liver might participate in the regulation of mitochondrial energy metabolism-associated proteins, such as cytochrome oxidase and ATP synthase, as well as other nuclear and cytosolic proteins, to harmonize energy supply and pathophysiological cell processes, e.g., proliferation and differentiation [10, 24].

The process of hepatocyte steatosis is the key to the onset of NAFLD, which is related to the regulation of energy metabolism and changes in the expressions of genes that control lipid metabolism, both of which might be associated with POP. In the present study, both POP mRNA and protein levels increased along with the steatosis of L02 cells, which suggests a non-negligible role of POP in hepatocyte steatosis. Indeed, our results revealed that POP inhibition by the specific inhibitor S17092 significantly attenuated lipid accumulation in the FFA-treated L02 cells. As previously mentioned, POP inhibition in the liver resulted in predominant changes in the mitochondrial oxidordeuctase/transporter, which might affect ATP synthesis via proteins such as cytochrome C oxidase and ATP synthase, and this could be essential to steatotic hepatocytes with POP levels were increased as we have demonstrated here [10, 24]. Further research is required to determine whether or how POP participates in hepatocyte steatosis via affecting mitochondrial function.

Increased expression of endogenous adipogenic genes is another reason for hepatocyte steatosis. Our research found that after POP inhibition, the expression levels of FASN and SREBP-1c, the key genes for the synthesis of endogenous long-chain fatty acids, were significantly decreased. Additionally, the expression level of PPAR-γ, which is a key gene in the regulation of lipid metabolism, was also decreased. Although the mechanism by which POP regulates these lipid metabolism-associated genes remains unclear, it could occur via the nuclear translocation of POP, which can directly regulate adipogenic gene expression-related proteins. This hypothesis is supported by other studies that demonstrated that translocation of POP into the nucleus in proliferating cells and that the inhibition of POP affects proteins involved in nucleic acid binding [10, 25].

Autophagy is linked to hepatocyte steatosis. Autophagy reduces intracellular lipid droplets by enclosing them and fusing them with lysosomes for degradation [26, 27]. Indeed, our results demonstrated that LC3B II, a bioprotein marker of autophagy, was significantly increased in the L02 cells that were treated with FFA for 24 h, which implies enhanced autophagy and is in agreement with a previous study [26]. Nonetheless, POP inhibition did not further increase LC3B II levels in the FFA-treated L02 cells, which suggests that enhanced autophagy may not be responsible for ameliorated lipid accumulation after short-term POP inhibition in FFA-treated L02 cells.

This experiment has some limitations. First, we cannot rule out a non-specific binding of this inhibitor. It should be noted that the concentrations of S17092 used in our study were much higher than the reported Ki value (about 1.5 nM) for purified human POP[15]. An explanation for this is that, in live cells, the actual concentration of S17092 needed for POP inhibition is affected by other factors, such as cell permeability, and intracellular inhibitor-enzyme binding affinity[15]; Notably, S17092 at the level of μM did not significantly affect activity of a wide range of peptidases, such as dipeptidylaminopeptidase IV, aminopeptidases B and M, or angiotensin-converting enzyme [15, 28] Second, the biological function of POP is rather complicated and is not restricted to its enzyme activity. Future experiments involving the manipulation of the POP protein by overexpression and silencing *in vitro* and *in vivo* will be helpful for supporting our results. Third, this experiment was rather descriptive, and further study is needed to further elucidate the underlying mechanism.

In general, this is the first experiment to suggest an important role of POP in hepatocytes steatosis and possibly NAFLD and may provide insight into the biological function of POP in the liver. Both the pathophysiological roles and actions of POP in hepatocytes are likely to be more complex than currently thought, and these roles require further clarification, especially with *in vivo* experiments.

## Supporting Information

S1 Fig Cytotoxicity of the POP inhibitor S17092 to L02 cells. (A-B) L02 cells were treated with 0.026–130 μM S17092 for 24 (A) or 48 h (B), and cytotoxicity was then evaluated by the supernatant LDH activity assay.
(TIF)
