# Supplementary figures and images for: Prolyl Oligopeptidase Inhibition Attenuates Steatosis in the L02 Human Liver Cell Line
Source: PLoS One. 2016 Oct 19;11(10):e0165224. doi: 10.1371/journal.pone.0165224 (PMC5070736; doi:10.1371/journal.pone.0165224)

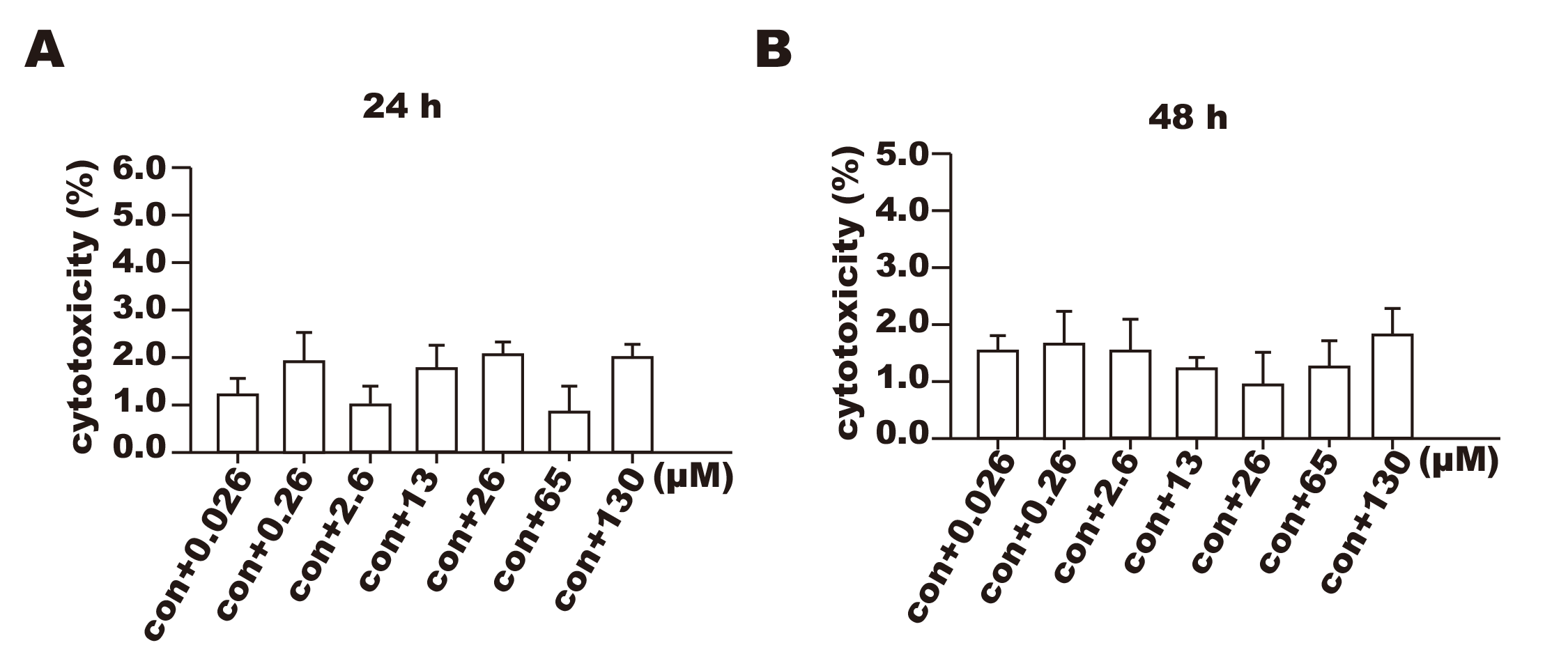

Supplement: S1 Fig — (A-B) L02 cells were treated with 0.026–130 μM S17092 for 24 (A) or 48 h (B), and cytotoxicity was then evaluated by the supernatant LDH activity assay. (TIF) [file pone.0165224.s001.tif]
